# A milder form of molybdenum cofactor deficiency type A presenting as Leigh's syndrome-like phenotype highlighting the secondary mitochondrial dysfunction: a case report

**DOI:** 10.3389/fneur.2023.1214137

**Published:** 2023-09-15

**Authors:** Montaha Almudhry, Asuri N. Prasad, C. Anthony Rupar, Keng Yeow Tay, Suzanne Ratko, Mary E. Jenkins, Chitra Prasad

**Affiliations:** ^1^London Health Sciences Centre, London, ON, Canada; ^2^Department of Neuroscience, King Fahad Specialist Hospital, Dammam, Saudi Arabia; ^3^Department of Pediatrics, Western University, London, ON, Canada; ^4^Department of Clinical Neurological Sciences, Western University, London, ON, Canada; ^5^Department of Biochemistry, Western University, London, ON, Canada; ^6^Department of Medical Imaging, Western University, London, ON, Canada

**Keywords:** *MOCS1*, dystonia, molybdenum cofactor deficiency, Leigh-like phenotype, stroke

## Abstract

**Background:**

Molybdenum cofactor deficiency (MoCD) (OMIM**#** 252150) is an autosomal-recessive disorder caused by mutations in four genes involved in the molybdenum cofactor (MOCO) biosynthesis pathway.

**Objectives:**

We report a milder phenotype in a patient with *MOCS1* gene mutation who presented with a Leigh-like presentation.

**Case report:**

We present the case of a 10-year-old boy who was symptomatic at the age of 5 months with sudden onset of dyskinesia, nystagmus, and extrapyramidal signs following a febrile illness. Initial biochemical, radiological, and histopathological findings a Leigh syndrome-like phenotype; however, whole-exome sequencing detected compound heterozygous mutations in *MOCS1* gene, c.1133 G>C and c.217C>T, confirming an underlying MoCD. This was biochemically supported by low uric acid level of 80 (110–282 mmol/L) and low cystine level of 0 (3–49), and a urine S-sulfocysteine at 116 (0–15) mmol/mol creatinine. The patient was administered methionine- and cystine-free formulas. The patient has remained stable, with residual intellectual, speech, and motor sequelae.

**Conclusion:**

This presentation expands the phenotypic variability of late-onset MoCD A and highlights the role of secondary mitochondrial dysfunction in its pathogenesis.

## Introduction

The molybdenum cofactor (MOCO) is an unstable pterin formed through complex biosynthesis processes; it plays a crucial role in the activity of molybdoenzymes, such as sulfite oxidase, xanthine dehydrogenase, and aldehyde oxidase, which are MOCO-dependent and function in the metabolism of xanthine and the catabolism of sulfur-containing amino acids. MOCO is synthesized in a multi-step reaction by *MOCS1, MOCS2, MOCS3*, and gephyrin (*GPHN*), which are the four genes identified in the Mo cofactor synthesis pathway. The lack of molybdenum cofactors causes functional deficiencies in the dependent enzymes, which are involved in sulfite detoxification and purine metabolism (1, 2).

Early-onset MoCD carries a poor prognosis; however, supplementation with cPMP (cyclic pyranopterin monophosphate) could improve the survival of patients with MoCD type A deficiency (3, 4).

There are only a few reports on patients with atypical late-onset MoCD. These highlight variable neurological and ocular presentations and radiological findings (5, 6). Typical Leigh syndrome-like findings represent an expanding heterogenic mitochondrial disease phenotype in early infancy, with psychomotor delay, encephalopathy, movement disorders, and breathing irregularities. Patients with Leigh syndrome share common neuroradiological features, including asymmetrical changes in the basal ganglia, brainstem, and cerebellum (7).

Here, we report an atypical, milder presentation of MoCD in an infant with a Leigh syndrome-like phenotype and the long-term follow-up.

## Case

The proband is a 10-year-old boy who was conceived through *in vitro* fertilization with a donor's oocyte and father's sperm. The pregnancy was uncomplicated. Delivery proceeded at term via the cesarean section because of failure to progress. The infant did not require resuscitation and was discharged home soon after birth.

At the age of 5 months, he developed a febrile illness associated with a sudden onset of bilateral continuous involuntary hyperkinetic movements involving his extremities, tilting his head to the right, horizontal nystagmus, and difficulty latching on (during breastfeeding) along with repetitive tongue thrusting. The patient's symptoms improved transiently for 2 days, followed by the reemergence of ocular nystagmoid movements along with involuntary movements involving the left hemibody with reduced tone on the right side. Examination confirmed the presence of dyskinetic movements involving the limbs along with nystagmoid eye movements and orolingual dyskinesis.

Prior to hospitalization, the infant met all age-appropriate milestones, including smiling, babbling, reaching, and sitting with support. However, during the acute illness, developmental regression with loss of motor skills was noted. Consanguinity status remained unknown, with no history of developmental delay, regression, or metabolic or neurological conditions on the paternal side.

**Figure 1:**
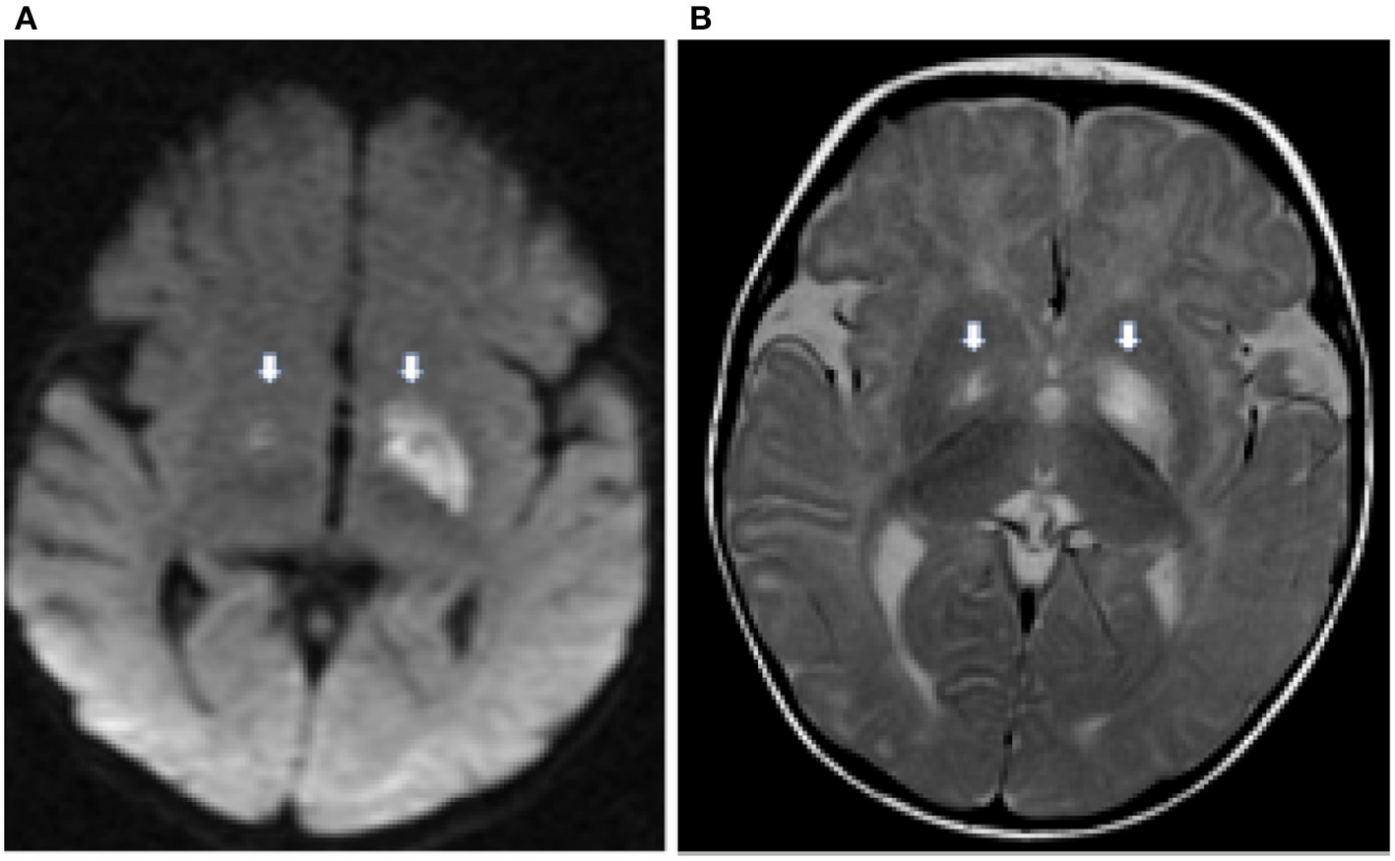
Axial diffusion weighted (DWI) image **(A)** demonstrates hyperintensity in the globus pallidus bilaterally (thick white arrows) representing cytotoxic edema from a recent infarct. There is corresponding hyperintensity on the T2 weighted image **(B)**.

**Figure 2:**
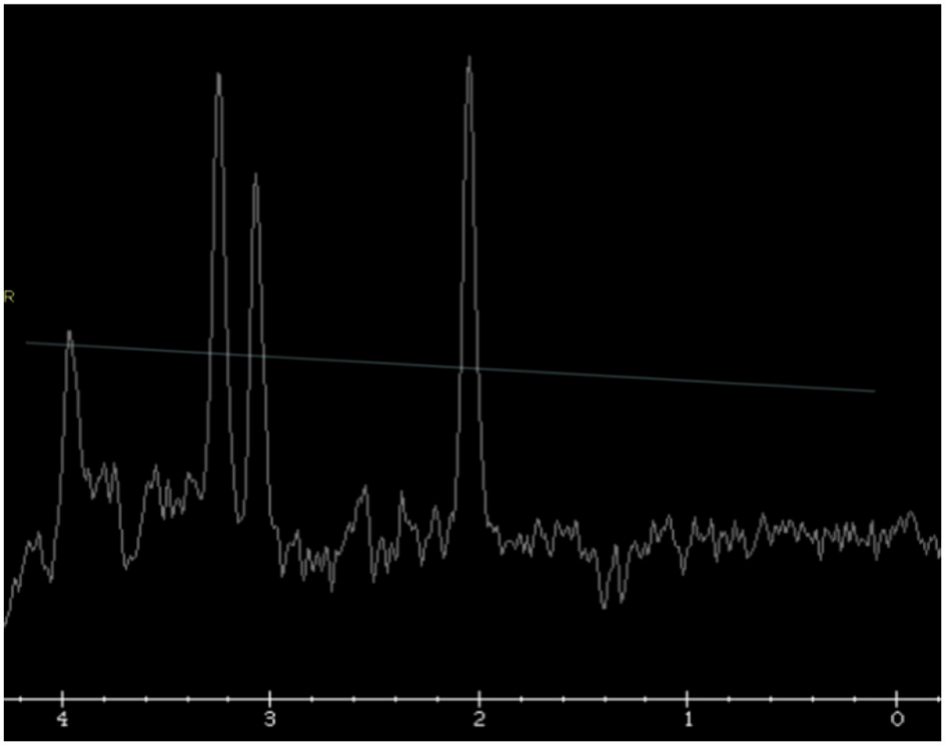
Single voxel MR spectroscopy in the left globus pallidus demonstrates a small, inverted lactate doublet at 1.3 ppm (thin white arrow).

MRI resonance imaging of the brain revealed the presence of asymmetrical high-intensity signal associated with diffusion restriction in the bilateral globus pallidus, suggesting metabolic stroke (Figures 1A, B). MR spectroscopy confirmed the presence of lactate peak in the left globus pallidus. These findings suggested an initial clinical diagnosis of Leigh syndrome (Figure 2).

The CSF amino acid evaluation showed slightly elevated alanine levels; however, the rest of the metabolic workup, including plasma amino acids, acylcarnitine profile, and urine organic acids, was normal. The results of the pyruvate dehydrogenase and pyruvate carboxylase assays were normal. Respiratory chain studies revealed reduced activity of complex II as well as very scant ragged red fibers in the muscle biopsy. These findings were again suggestive of a mitochondrial disorder. The patient was discharged from hospital with an empirical mitochondrial cocktail containing carnitine, biotin, thiamine, riboflavin, and ubiquinone. Upon discharge, the patient's involuntary movements were in remission with good feeding skills but with reduced right-sided spontaneous movement and strength.

Genetic testing of the mitochondrial genome and full mitochondrial nuclear gene panel yielded negative results. Selective sequencing of *AARS2* and *SDHA* yielded normal results. Whole-exome sequencing detected compound heterozygous mutations in *MOCS1* gene. The variant p. R378P (c. 1133 G>C) was inherited from the father. The second variant, p.R73W (c.217C>T), was likely inherited from the biological mother (oocyte donor); this variant has been previously reported in individuals with MoCD (8). This variant was observed in 9/25,778 (0.035%) non-Finnish European individuals. A missense variant at the same residue (R73Q) was reported in individuals with MoCD; this supports the functional importance of this protein region. Both variants were interpreted as variants of unknown significance due to the lack or insufficiency of data in a large population cohort. However, both variants encode non-conserved amino acid substitutions that are likely to impact the secondary protein structure because these residues differ in polarity, charge, and size. *In silico* analyses of both variants, including protein predictors and evolutionary conservation, indicate a deleterious effect. Biochemical testing for MoCD detected a low uric acid level of 80 (111–282 μmol/L) and a low cystine level of 0 (3–49) and 4 (7–40) umols per liter, which remained low throughout the entire course, ranging between 5 and 9 (normal 17–43 umol/L). On the other hand, CSF amino acids were done once, and the cystine in the CSF was below detectable limits. Moreover, the urine S-sulfocysteine was high at 116 (0–15) mmol/mol creatinine and 163 (0–50) mmol/mol creatinine on two separate occasions. These findings on biochemical testing were consistent with the diagnosis of molybdenum cofactor deficiency.

The child was started on X-Met X-Cys Maxamaid, a methionine- and cystine-free formula; however, he was unable to tolerate the total prescribed amount and remained otherwise on protein-restricted diet. At 9 years of age, his weight remains low at 25.7 kg (9th centile), height at 130 cm (10th centile), and BMI at 21st centile. During follow-up, parents reported neurological decompensation during inter-current illnesses. He developed signs of dystonic posturing superimposed right spastic hemiparesis, along with significant dysarthria. It was observed that his dystonia worsened with movement, walking, and running. He could not fully extend his arm and hand, which were held in flexion and the hand in a fisted position, while his leg was held in extension with plantarflexion and inversion at the ankles. He was evaluated at the pediatric movement disorders clinic, and levodopa/carbidopa 100/25 mg was begun and gradually increased to 100 mg/25 mg three times daily for his dystonia. Due to intermittent GI discomfort, further dose increments were not pursued, and subsequently, amantadine was added as an adjunct to L-dopa. The two medications together have helped him to loosen up his dystonic posturing and his speech improved.

The child remains globally delayed in gross motor, fine motor, and speech although he continues to maintain slow progress. He is presently able to walk independently with a right hemiparetic, hemidystonic gait. He also enjoys horseback riding, where he can maintain his balance perfectly. Fine motor skills have been challenging although he is able to handle playing with Lego parts. His expressive language abilities are improving, and he is presently able to put words together to make grammatical sentences, but speech remains slurred and not intelligible to strangers. By next fall, the patient will be in grade 5, attending a special school where accommodations have been tailored to his neurodevelopmental disabilities, cognitive impairments, and dietary restrictions.

His current medications include levodopa/carbidopa 100/25 one tablet TID, amantadine 25 mg q a.m., CoQ10 hundred 50 mg BID, riboflavin 100 mg BID, carnitine 150 mg TID, and thiamine 150 mg BID.

## Discussion

Molybdenum is a trace element that is actively inserted into pterin to form an enzyme cofactor (MOCO), which plays a role in the functioning of four catalytic enzymes: sulfite oxidase, xanthine dehydrogenase, aldehyde oxidase, and the mitochondrial amidoxime reducing component (mARC). MOCO biosynthesis is a multi-step process that begins with the conversion of GTP into cyclic pyranopterin monophosphate (cPMP), which is the first and most stable MOCO intermediate. *MOCS1A* and *MOCS1B* encode proteins that catalyze the conversion of GTP to cPMP. The second step involves transferring a sulfur group from cPMP to MPT using the MPT synthase enzyme encoded by *MOCS2A* and *MOCS2B*. Following this step, the MPT synthase should be resulfurated by the enzyme, MPT synthase sulfurase, encoded by *MOCS3*, for reactivation. Molybdate is transferred to MPT by molybdenum insertase. The final step involves the transfer of MPT-AMP from the G to the E domain to create MOCO (Supplementary Figure 2) (1, 9, 10).

Biallelic pathogenic mutations in any of the genes involved in the MOCO biosynthesis pathway can translate clinically to MoCD (11, 12). Two distinct presentations have been identified: early- and late-onset neonatal encephalopathy (5, 13). Early-onset molybdenum cofactor deficiency (MoCD) is an autosomal-recessive disease that typically manifests as a progressive hypoxic ischemic encephalopathy (HIE)-like phenotype in neonates, with microcephaly, refractory epilepsy, psychomotor retardation, exaggerated startle response, autonomic dysfunction, and progressive pyramidal and extrapyramidal signs. In addition, this disease causes extra-CNS manifestations, such as dislocated lenses, feeding difficulties, obesity, and renal calculi (14). Subcortical cystic encephalomalacia, calcification of the basal ganglia and/or the thalamus, and atrophy of the basal ganglia are common neuroimaging findings. The biochemical profile revealed reduced levels of uric acid in the plasma and elevated levels of purine and sulfite metabolites in the urine, reflecting a deficiency in MOCO-dependent enzymatic activity (15).

Late-onset patients present with a variety of symptoms ranging from catastrophic acute presentations to mild symptoms. Scelsa et al. (16) reviewed and reported 13 patients with the mild phenotype. Patients typically develop acute-onset focal pyramidal or extrapyramidal deficits after their first birthday often because of an intercurrent illness or trivial injury. Recurrent physiological stress can lead to decompensation and focal deficits. Behavioral problems are among the common features of these patients, along with mild cognitive deficiencies and language delay/regression. Unlike neonatal-onset diseases, later-onset epilepsy is neither frequent nor pharmacoresistant. Various ocular manifestations, including spherophakia, glaucoma, and retinal attachment, have been reported. However, lens subluxation/dislocation remains the most consistent ocular finding. In addition, dysmorphic features could be a part of the late-onset phenotype, including one reported patient with Marfanoid habitus. The biochemical profiles of the late-onset form are marked by the same metabolic derangements as that of the early-onset form, including elevated S-sulfocysteine and low uric acid levels. Nevertheless, brain scans show changes limited to the basal ganglia and dentate nucleus in delayed presentations, as opposed to the extensive MRI abnormalities in the neonatal form (6, 17, 18).

The index case presented with a constellation of findings at 5 months of age suggesting a Leigh-like phenotype with an abrupt onset of dyskinetic movement, loss of previously acquired milestones, and hypotonia. Brain MRI showed bilateral asymmetrically involved globi pallidi with an inverted lactate peak at 1.3 PPM in the MRS study. Alanine levels were elevated in the plasma. Electron transfer chain function analysis documented reduced activity of complex II along with illustrating ragged red fiber pattern in muscle biopsy. These findings indicate the possibility of an underlying mitochondrial cytopathy that explains this presentation. Nevertheless, whole-exome sequencing and subsequent biochemical testing confirmed the diagnosis of MoCD type A. A similar initial presentation of Leigh-like phenotype was previously reported in a patient with MoCD type B (19), with subsequent molecular confirmation of MoCD.

The later emergence of dystonia and gait disturbance presented a treatment challenge for the treating team. The symptoms of dystonia were superimposed what was assumed to be a small vessel stroke involving the basal ganglia. The neurological examination did not have all the features of vascular Parkinsonism, but the involvement of basal ganglia in association with the clinical syndrome provided the basis for introduction of the trial of L-dopa/carbidopa which was of modest benefit (20). However, since the child developed gastrointestinal symptoms which limited the use of incremental doses of L-dopa/carbidopa, amantadine was added as an adjunct. Amantadine is a non-competitive NMDA receptor antagonist shown to enhance benefits and improve motor complications in patients with when given adjunctively with levodopa (21). The combination proved to be extremely helpful in management in this case by significantly improving both abnormal posturing and tone associated with dystonia and speech.

Under a variety of heritable and acquired conditions, secondary attenuation of mitochondrial function can affect the dynamics of mitochondria, affecting their ability to produce ATP without any primary underlying inborn defects in genes that regulate the OXPHOS pathway. Methylmalonic and propionic aciduria, fatty acid oxidation disorders, disorders of purine/pyrimidine synthesis, and urea cycle disorders are among the many inborn errors of metabolism in which secondary mitochondrial disease is part of the clinical and biochemical phenotypes (22, 23).

MOCO-dependent sulfite oxidase is located in the mitochondrial intermembrane space and is involved in the final catabolic step involving sulfur-containing amino acids. It catalyzes the oxidation of sulfite to sulfate (${\text{SO}}_{4}^{2-}$). Sulfite oxidase can be deficient due to a mutation in the *SUOX* gene or secondary to a MOCO biosynthesis defect. Both result in a clinically and radiologically indistinguishable phenotype; however, they can be differentiated on biochemical and genetic basis. Deficiency in sulfite oxidase activity leads to elevated sulfite levels, which impairs mitochondrial function and leads to excitotoxic neuronal injury and cell death (4). During the culture of embryonic fibroblasts from SO-deficient mice in the galactose-containing medium, impaired ATP production and reduced growth rates were observed; these were directly linked to elevated ${\text{SO}}_{3}^{-2}$ levels (24, 25). This sulfite accumulation could be the basis for stroke-like episodes in these patients (16).

Observing a Leigh-like phenotype and mitochondrial dysfunction in both animal models and patients with MoCD/isolated sulfite deficiency suggests that secondary mitochondrial dysfunction plays a role in the etiopathogenesis of patients with MoCD. Identifying these patients early could permit the initiation of cPMP, special diet, and placement on a mitochondrial cocktail to improve overall survival and help support mitochondrial function (26).

## Conclusion

This case report describes a late-onset MoCD type A patient presenting with a Leigh-like phenotype following a febrile illness. This increases the phenotypic spectrum of this disorder and highlights the secondary mitochondrial dysfunction in this disease. These findings could have potential therapeutic implications for affected individuals.

## Data availability statement

The original contributions presented in the study are included in the article/Supplementary material, further inquiries can be directed to the corresponding author.

## Ethics statement

Ethical review and approval was not required for the study on human participants in accordance with the local legislation and institutional requirements. Written informed consent to participate in this study was provided by the participants' legal guardian/next of kin. Written informed consent was obtained from the minor(s)' legal guardian/next of kin for the publication of any potentially identifiable images or data included in this article.

## Author contributions

MA carried out the data review and drafted the manuscript. CP and AP are the primary treating physicians who also guide through reviewing and editing the manuscript. MJ and SR are part of the treatment team and helped revise their corresponding parts of the manuscript. KT participated in choosing and commenting on the radiological studies. CR helped with molecular interpretation. All authors read and approved the manuscript.

## References

[1] Huijmans JGM Schot R de Klerk JBC Williams M de Coo RFM Duran M. Molybdenum cofactor deficiency: identification of a patient with homozygote mutation in the MOCS3 gene. Am J Med Genet Part A. (2017) 173:1601–6. 10.1002/ajmg.a.38240 28544736

[2] Martínez MCA Cazorla E Cánovas E Anniuk K Cores AE Serrano AM. Molybdenum cofactor deficiency: mega cisterna magna in two consecutive pregnancies and review of the literature. Appl Clin Genet. (2020) 13:49–55. 10.2147/TACG.S239917 32099439 PMC6999763

[3] Zaki MS Selim L EL-Bassyouni HT Issa MY Mahmoud I Ismail S. Molybdenum cofactor and isolated sulphite oxidase deficiencies: clinical and molecular spectrum among Egyptian patients. Eur J Paediatr Neurol. (2016) 20:714–22. 10.1016/j.ejpn.2016.05.011 27289259 PMC4993451

[4] Nagappa M Bindu PS Taly AB Sinha S Bharath RD. Child neurology: molybdenum cofactor deficiency. Neurology. (2015) 85:e175–8. 10.1212/WNL.0000000000002194 26644055

[5] Vijayakumar K Gunny R Grunewald S Carr L Chong KW Devile C. Clinical neuroimaging features and outcome in molybdenum cofactor deficiency. Pediatr Neurol. (2011) 45:246–52. 10.1016/j.pediatrneurol.2011.06.006 21907887

[6] Yan W Huang L Sun L Ding X. Ocular characteristics of a 6-year-Old boy with molybdenum cofactor deficiency type B. Am J Ophthalmol Case Reports. (2022) 27:101586. 10.1016/j.ajoc.2022.101586 35692435 PMC9178334

[7] Lee JS Yoo T Lee M Lee Y Jeon E Kim SY. Genetic heterogeneity in Leigh syndrome: Highlighting treatable and novel genetic causes. Clin Genet. (2020) 97:586–94. 10.1111/cge.13713 32020600

[8] Reiss J Christensen E Kurlemann G Zabot MT Dorche C. Genomic structure and mutational spectrum of the bicistronic MOCS1 gene defective in molybdenum cofactor deficiency type A. Hum Genet. (1998) 103:639–44. 10.1007/s004390050884 9921896

[9] Mendel RR. The molybdenum cofactor. J Biol Chem. (2013) 288:13165–72. 10.1074/jbc.R113.455311 23539623 PMC3650355

[10] Mendel RR Leimkühler S. The biosynthesis of the molybdenum cofactors. J Biol Inorg Chem. (2015) 20:337–47. 10.1007/s00775-014-1173-y 24980677

[11] Reiss J. Genetics of molybdenum cofactor deficiency. Hum Genet. (2000) 106:157–63. 10.1007/s004399900223 10746556

[12] Reiss J Lenz U Aquaviva-Bourdain C Joriot-Chekaf S Mention-Mulliez K Holder-Espinasse M. point mutation leading to molybdenum cofactor deficiency. Clin Genet. (2011) 80:598–9. 10.1111/j.1399-0004.2011.01709.x 22040219

[13] Misko AL Liang Y Kohl JB Eichler F. Delineating the phenotypic spectrum of sulfite oxidase and molybdenum cofactor deficiency. Neurol Genet. (2020) 6:1–8. 10.1212/NXG.0000000000000486 32802950 PMC7371372

[14] Bayram E Topcu Y Karakaya P Yis U Cakmakci H Ichida K. Molybdenum cofactor deficiency: review of 12 cases (MoCD and review). Eur J Paediatr Neurol [Internet]. (2013) 17:1–6. 10.1016/j.ejpn.2012.10.003 23122324

[15] Atwal PS Scaglia F. Molybdenum cofactor deficiency. Mol Gen Metabol. (2016) 117:1–4. 10.1016/j.ymgme.2015.11.010 26653176

[16] Scelsa B Gasperini S Righini A Iascone M Brazzoduro VG Veggiotti P. Mild phenotype in Molybdenum cofactor deficiency: a new patient and review of the literature. Mol Genet Genomic Med. (2019) 7:1–9. 10.1002/mgg3.657 30900395 PMC6565584

[17] Sharawat IK Saini L Singanamala B Saini AG Sahu JK Attri SV. Metabolic crisis after trivial head trauma in late-onset isolated sulfite oxidase deficiency: report of two new cases and review of published patients. Brain Dev. (2020) 42:157–64. 10.1016/j.braindev.2019.11.003 31806255

[18] Misko A Mahtani K Abbott J Atwal P. Molybdenum cofactor deficiency summary genetic counseling. Gene Rev. (2021) 3:1–20.

[19] Pediatr CJ. 以 Leigh 样综合征为表现的钼辅因5缺乏症 B 型一例并文献复习. (2021) 59:119–24. 10.3760/cma.j.cn112140-20200911-00866 33548958

[20] Siniscalchi A Gallelli L Labate A Malferrari G Palleria C De Sarro G. Post-stroke movement disorders: clinical manifestations and pharmacological management. Curr Neuropharmacol. (2012) 10:254–62. 10.2174/157015912803217341 23449883 PMC3468879

[21] Sy MAC Fernandez HH. Dystonia and leveraging oral pharmacotherapy. J Neural Transm. (2021) 128:521–9. 10.1007/s00702-021-02339-7 33877451

[22] Khajuria K Khajuria V Sawhney V. Secondary mitochondrial dysfunction. Int J Pharm Pharm Sci. (2021) 13:14–9. 10.22159/ijpps.2021v13i3.40335

[23] Niyazov DM Kahler SG Frye RE. Primary mitochondrial disease and secondary mitochondrial dysfunction: importance of distinction for diagnosis and treatment. Mol Syndromol. (2016) 7:122–37. 10.1159/000446586 27587988 PMC4988248

[24] Mellis AT Roeper J Misko AL Kohl J Schwarz G. Sulfite alters the mitochondrial network in molybdenum cofactor deficiency. Front Genet. (2021) 11:1–10. 10.3389/fgene.2020.594828 33488670 PMC7817995

[25] Glänzel NM Grings M da Rosa-Junior NT de Carvalho LMC Mohsen AW Wipf P. The mitochondrial-targeted reactive species scavenger JP4-039 prevents sulfite-induced alterations in antioxidant defenses, energy transfer, and cell death signaling in striatum of rats. J Inherit Metab Dis. (2021) 44:481–91. 10.1002/jimd.12310 32882059 PMC8039837

[26] Hitzert MM Bos AF Bergman KA Veldman A Schwarz G Santamaria-Araujo JA. Favorable outcome in a newborn with molybdenum cofactor type A deficiency treated with cPMP. Pediatrics. (2012) 130:1005–10. 10.1542/peds.2011-3330 22987873

