# Supplementary material for: A milder form of molybdenum cofactor deficiency type A presenting as Leigh's syndrome-like phenotype highlighting the secondary mitochondrial dysfunction: a case report
Source: Front Neurol. 2023 Sep 15;14:1214137. doi: 10.3389/fneur.2023.1214137 (PMC10542394; doi:10.3389/fneur.2023.1214137)
Supplement: Supplementary file 1 [file Data_Sheet_1.docx]

Supplementary Material

A Milder form of Molybdenum cofactor deficiency type A presenting as Leigh's syndrome-like phenotype highlighting the secondary mitochondrial dysfunction: a case report

Montaha Almudhry^a,e^, Asuri N. Prasad^a,c,f^, C. Anthony Rupar^a,b,c^,, Keng Yow Tay^a,d^, Suzanne Ratko^a,c^, Mary E Jenkins^a,f^, Chitra Prasad^a,c^

^a^London Health Sciences Centre, London, Ontario, Canada

^b^Department of Biochemistry, Western University, London, Ontario, Canada

^c^Department of Pediatrics, Western University, London, Ontario, Canada

^d^Department of Medical Imaging, Western University, London, Ontario, Canada

^e^Department of Neuroscience, King Fahad specialist hospital, Dammam, Saudi Arabia

^f^Department of Clinical Neurological sciences, Western University, London, Ontario, Canada

Correspondence to: Dr. Asuri N. Prasad MBBS, MD, FRCPC, FRCPE, FAES

Address: Professor Pediatrics and Clinical Neurosciences

Department of Paediatrics, Children’s Hospital, London Health Sciences Centre, 800 Commissioners Road East, London, Ontario, Canada N6A5W9

E-mail address: narayan.prasad@lhsc.on.ca

**Supplementary Figures:**

Figure 1b

Figure 1a


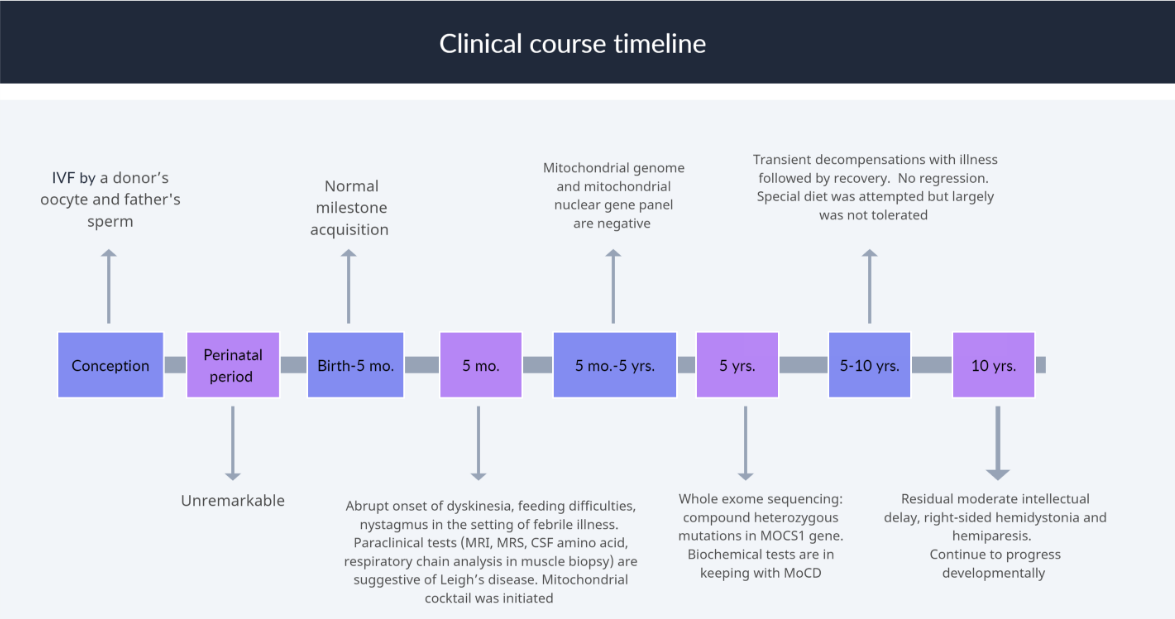


Supplementary Figure 1: Clinical course timeline


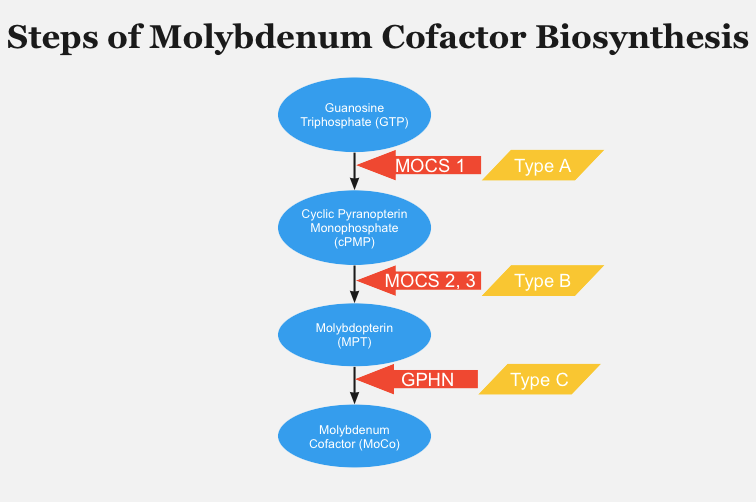


**Supplementary Figure 2.** Molybdenum cofactor biosynthesis
